# Estimated Prevalence of Glaucoma in South Korea Using the National Claims Database

**DOI:** 10.1155/2016/1690256

**Published:** 2016-05-09

**Authors:** Sang Jin Seo, Yun Ha Lee, Sang Yeop Lee, Hyoung Won Bae, Samin Hong, Gong Je Seong, Chan Yun Kim

**Affiliations:** Department of Ophthalmology, Severance Hospital, Institute of Vision Research, Yonsei University College of Medicine, Seoul 120-752, Republic of Korea

## Abstract

*Purpose*. To estimate the prevalence of glaucoma and costs associated with glaucoma care in South Korea between 2008 and 2013 using the Korean national claims database.* Design*. Retrospective cross-sectional study from a national claims database.* Methods*. Patients who were diagnosed with glaucoma between 2008 and 2013 were retrospectively identified in the national claims database using glaucoma diagnostic codes. For each year, the prevalence of glaucoma and direct medical costs associated with glaucoma care were estimated.* Result*. The prevalence of glaucoma in patients ≥40 years of age increased from 0.79% in 2008 to 1.05% in 2013. The number of patients with glaucoma increased by 54% between 2008 and 2013 (9% average annual increase). The prevalence of glaucoma increased with age and was higher in males than in females. The cost to care for glaucoma patients increased from $16.5 million in 2008 to $29.2 million in 2013, which translated into an 81% increase over the 6 years examined (12.7% average annual increase).* Conclusion*. The estimated prevalence and socioeconomic burden of glaucoma have steadily increased each year in South Korea. Nevertheless, many glaucoma patients remain undiagnosed in the present study using national claims database.

## 1. Introduction

Glaucoma is a chronic eye disease that results in irreversible optic nerve damage, which causes severe vision loss and blindness. It is the second leading cause of blindness worldwide and one of the leading causes of irreversible blindness [1]. The estimated number of people around the world with glaucoma was 64.3 million in 2013, and this number is expected to increase to 111.8 million in 2040. The increasing trend in the number of patients with glaucoma will continue because of increased life expectancy. In recently published systematic reviews, the glaucoma prevalence in subjects >40 years of age was estimated to be 3.54% (95% confidence interval [CI]: 2.09–5.82) globally and 3.40% (95% CI: 2.26–5.02) in Asia [2].

Most studies examining the prevalence of glaucoma have been population-based, but these may be limited in determining glaucoma prevalence in real world because of differences in setting of glaucoma diagnosis between clinical practice and population-based studies. In additional, population-based studies varied widely in terms of the eye examination methods and case definitions used [3].

Korean National Health Insurance (NHI) covers 50 million people in South Korea, which represents approximately 97% of the Korean population. Therefore, we may be able to use this database to obtain accurate statistics on glaucoma in South Korea. These national claims database provides unique real-world data from a large, complete, and unselected population, providing important information about all types of glaucoma disease [3].

In the current study, we estimated the prevalence of glaucoma between 2008 and 2013 in Korea using the national health claims database. This prevalence was then compared to estimates made with previous population-based studies. The direct economic burden of glaucoma in Korea from 2008 to 2013 was also investigated.

## 2. Materials and Methods

All study conduct adhered to the tenets of the Declaration of Helsinki. The Institutional Review Board of Yonsei University Health System determined that this study qualified for exempt status (IRB permit number: 2014-2520-001). We used data that was recorded in the national health claims database between 2008 and 2013. Data were obtained from the Health Insurance Review and Assessment (HIRA) service of Korea (http://www.hira.or.kr/), which provided deidentified data for this study. The HIRA is a governmental agency overseen by the Korean Ministry of Health and Welfare that examines and evaluates medical expenses of all citizens (approximately 50 million people; 96.6% of the population) covered by Korean NHI, the compulsory social insurance, and Medical Aid (approximately 3.4% of the population). The HIRA database contains payment records from all medical facilities in South Korea and includes information from 5-6 million inpatient visits each year at approximately 1,100 hospitals and 25,000 private clinics [4]. The database contains data on diagnoses, procedures, prescriptions, demographics, and direct medical costs. The HIRA database manages claims using the sixth edition of the Korean Classification of Disease (KCD-6), which is similar to the International Classification of Disease (ICD-10) system. The diagnostic codes for glaucoma are H40 (glaucoma) and H42 (glaucoma in other diseases classified elsewhere). The H40 code family includes diagnoses of glaucoma suspect (or ocular hypertension [OHT], H400), open angle glaucoma (OAG, H401, including normal tension glaucoma [NTG] and primary open angle glaucoma [POAG]), angle closure glaucoma (ACG, H402), secondary glaucoma (H403–406), other glaucoma (H408), and unspecified glaucoma (H409). The H42 code family includes diagnoses of glaucoma related to endocrine, nutritional, and metabolic diseases (H420). Surgical codes used to identify patients with glaucoma were those for nonpenetrating filtering surgery (S5040), iridotomy (S5041), filtering surgery (S5042), trabeculectomy (S5043), cyclophotocoagulation (S5044), cyclocryosurgery for glaucoma (S4055), trabeculotomy (S4057), sinusotomy (S4058), and glaucoma implant surgery (S5049).

Data from the HIRA database were used to estimate the size of the glaucoma population in South Korea. From all payment requests between 2008 and 2013, we extracted all requests for inpatient and outpatient services for patients >40 years old whose main or secondary cause for the visit was documented with an H40 or H42 KCD diagnosis code. Patients that were given an H400 diagnosis code (glaucoma suspect or OHT) were excluded from glaucoma prevalence estimations.

For the calculation of glaucoma care cost, we applied the annual KRW- (Korean Won-) USD (US dollar) exchange rate of each year and current KRW for each year was converted to constant KRW (2013) using an annual inflation rate of 3% (average increase in consumer price index over 6-year period in South Korea). In the glaucoma care cost, all payment requests for glaucoma diagnosis code were included such as diagnostic test, procedure, and prescriptions.

The annual prevalence of glaucoma was calculated as the number of people who were diagnosed with glaucoma divided by the number of people that received NHI coverage that year. The prevalence of glaucoma was based on patients >40 years of age and was examined by age and sex. The 95% CI of the prevalence rate was estimated based on a Poisson distribution. Linear trend analyses were performed using Mantel-Haenszel tests. All statistical analyses were performed using SPSS statistical software (ver. 19.0; SPSS, Inc., Chicago, IL, USA). Statistical significance was defined as a *P* value of <0.05.

## 3. Results

Among the general population that was at least 40 years old in South Korea, about 30% visited an ophthalmologist at least once a year. This totaled approximately 7.0 million people in 2008 and 9.6 million in 2013. The numbers of patients with glaucoma increased by approximately 9% each year (54.15% increase between 2008 and 2013) and in 2013, more than 270,000 Koreans visited the hospital for glaucoma. The total cost of glaucoma care in 2013 was over $29.8 million (USD) and increased by approximately 81% between 2008 and 2013. The cost of glaucoma care per person per year increased from approximately $93.6 in 2008 to $110 in 2013 (Table 1). The estimated prevalence of glaucoma had a significantly increasing trend between 2008 and 2013 (0.796% in 2008, 0.835% in 2009, 0.883% in 2010, 0.979% in 2011, 1.006% in 2012, and 1.056% in 2013, *P* < 0.001, Mantel-Haenszel test for linear trend, Table 2). The estimated prevalence of glaucoma suspects was 0.678% in 2008, 0.738% in 2009, 0.814% in 2010, 1.041% in 2011, 1.136% in 2012, and 1.206% in 2013. The prevalence of glaucoma among people who visited an eye clinic was 2.53% in 2008, 2.53% in 2009, 2.49% in 2010, 2.65% in 2011, 2.71% in 2012, and 2.82% in 2013 (Table 1).

The most common glaucoma subtype was OAG, which accounted for 56–62% of all glaucoma cases. The prevalence of OAG, including NTG, was 0.459% (57.6% OAG) in 2008, 0.475% (56.9% OAG) in 2009, 0.497% (56.3% OAG) in 2010, 0.574% (58.6% OAG) in 2011, 0.606% (60.2% OAG) in 2012, and 0.651% (61.6% OAG) in 2013. The proportion of patients with ACG was 8–12%. The prevalence of ACG was 0.093% (11.7% ACG), in 2008, 0.095% (11.4% ACG) in 2009, 0.1% (11.3% ACG) in 2010, 0.095% (9.7% ACG) in 2011, 0.090% (8.9% ACG) in 2012, and 0.090% (8.6% ACG) in 2013. The OAG/ACG ratio was 4.94 in 2008, 5.00 in 2009, 5.00 in 2010, 6.04 in 2011, 6.75 in 2012, and 7.21 in 2013 (Table 2).

**Figure 1:**
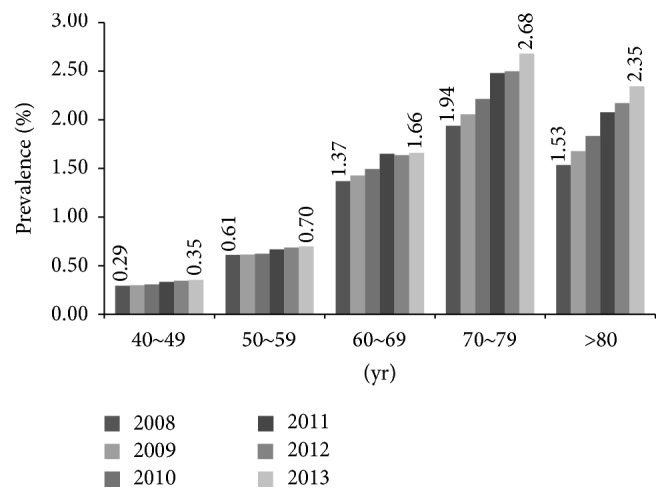
Prevalence of glaucoma in each age group. The prevalence of glaucoma significantly increased with age in Korean adults. The trend of increasing prevalence was statistically significant in older (>60 years) groups.

The prevalence of glaucoma increased with age. In 2013, the glaucoma prevalence was 2.35% in subjects >80 years old, 2.68% in subjects 70–79 years old, 1.66% in subjects 60–69 years old, 0.70% in subjects 50–59 years old, and 0.35% in subjects 40–49 years old. The prevalence of glaucoma increased annually in each age group but was significantly higher in older age groups (>60 years) than in younger ones (Figure 1). After adjusting for gender, the odds ratio (OR) of developing glaucoma was 1.616–1.759 with each decade increase in age (Table 3). Glaucoma prevalence was higher in males than in females during the time period examined (Table 2). After adjusting for age, males were more likely to have glaucoma than females (OR: 1.131–1.300, Table 3).

**Figure 2:**
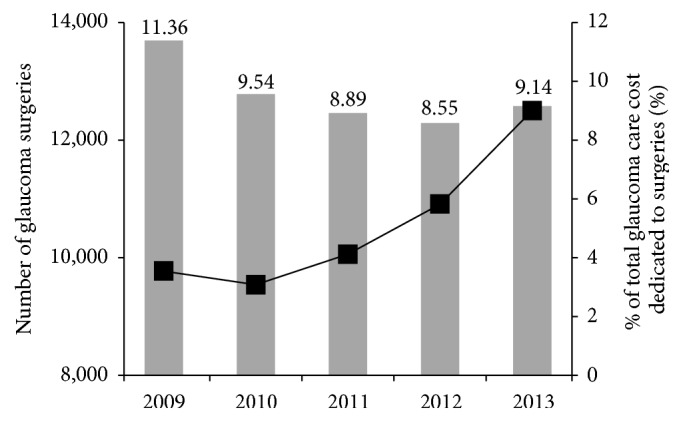
Trends in glaucoma surgery costs and number of procedures in Korea. The number of glaucoma surgeries performed (black squares) increased annually, but the proportion of total glaucoma care costs associated with glaucoma surgery (gray bars) decreased annually. Both trends were statistically significant.

The total number and cost of glaucoma surgeries increased annually, with 9773 glaucoma surgeries performed in 2009, 9541 surgeries performed in 2010, 10,061 surgeries performed in 2011, 10,912 surgeries performed in 2012, and 12,502 surgeries performed in 2013. The most common type of glaucoma surgery performed (including laser procedures) was iridotomy, which accounted for 54.9% of surgeries in 2009, 55.4% of surgeries in 2010, 54.4% of surgeries in 2011, 53.2% of surgeries in 2012, and 49.6% of surgeries in 2013. Trabeculectomy was the next most common surgery and accounted for 23.6% of surgeries in 2009, 22.6% of surgeries in 2010, 24.7% of surgeries in 2011, 28.1% of surgeries in 2012, and 32.1% of surgeries in 2013. The third most common surgery was glaucoma implant surgery, which accounted for 8.5% of surgeries in 2009, 9.9% of surgeries in 2010, 9.2% of surgeries in 2011, 9.1% of surgeries in 2012, and 8.1% of surgeries in 2013. The proportion of iridotomies decreased over time, while that of trabeculectomies increased each year. The cost for all glaucoma surgeries reached approximately $2.88 million in 2013 and the proportion of total glaucoma care costs that were related to glaucoma surgeries significantly decreased from 11.36% in 2009 to 9.14% in 2013 (Figure 2, Table 4).

## 4. Discussion

We assessed the estimated prevalence of glaucoma and the trend in glaucoma-associated figures in Korea over 6 years (2008 to 2013) using the national claims database. Advantages of using the national claims database over population surveys include its very large sample size and its more representative diagnostic patterns. Korean NHI is compulsory social insurance that covers 50 million Korean people (approximately 97% of population). It is more powerful than any other national claims database. Additionally, claims data are not regional, but nationwide, which provide more accurate estimates of disease prevalence in Korea. Because claims data are longitudinal, they can also be used to recognize trends over time in disease prevalence.

In the present study, the number of patients with glaucoma significantly increased each year (54% increase between 2008 and 2013). Tham et al. [2] estimated that the number of people worldwide with glaucoma will increase over 2013 levels by 18.3% in 2020 and by 74% in 2040, largely because of an increased life expectancy. In our study, the proportion of elderly patients (>60 years) in the Korean NHI database gradually increased from 14.28% in 2008 to 16.88% in 2013. This was not enough to explain the marked increase we observed in the incidence of glaucoma, which was higher than in previous studies [1, 2].

It is possible that the dramatic increase in people with glaucoma observed here does not reflect an increase in glaucoma prevalence. Rather, the glaucoma increase may reflect improvements in glaucoma detection, which may have occurred for several reasons. First, access to eye healthcare improved during the study period. Between 2008 and 2013, the number of eye clinics in Korea increased by 9% (1301 clinics in 2008 and 1418 clinics in 2013) and the number of ophthalmologists increased by 23% (determined from NHI database). The increase in refractive and cataract surgery also led to improvements in glaucoma detection in Korea. In almost all eye clinics, preoperative ophthalmic examinations include fundus and optic nerve examinations. Therefore, the higher surgical rate increased the chance of identifying patients with asymptomatic glaucoma. Second, the national health education and health promotion program has increased interest and awareness about health and disease in Korea. Additionally, the development of the internet and multimedia has improved disease awareness among the general population. Informing the community about glaucoma is an important step in promoting preventive ophthalmic care [5]. In Korea, news stories related to glaucoma significantly increased from 533 stories in 2008 to 1490 stories in 2013 (data obtained from the internet portal site, NAVER [http://www.naver.com/]). Additionally, the Korean national health check-up program does not include a fundus examination, but most hospitals have implemented programs that include fundus examinations as part of medical check-ups. Therefore, more fundus examinations were performed as the population received more recent medical check-ups, which subsequently resulted in more glaucoma diagnoses.

Several studies have reported the prevalence of glaucoma in East Asia and in South Korea. Kim et al. [6] found that the prevalence of glaucoma in rural populations over 50 years of age was 3.4% (95% CI: 2.1%–4.8%). The Namil Study Group performed a population-based survey of glaucoma in a rural area of central South Korea and found that the prevalence of glaucoma was 4.5% [7]. Similarly, the Tajimi Study Report [8, 9] reported a glaucoma prevalence of 5.0% (95% CI: 4.2%–5.8%) in central Japan and a systematic review reported a glaucoma prevalence of 2.26%–5.02% in Asia [2]. Although the prevalence of glaucoma among the population that visited an eye clinic was similar to other reports (2.53–2.82%), we found a much lower estimated prevalence of glaucoma among the total population (1.06%), as well as a lower POAG prevalence (0.65%), in 2013 based on the national claims database. We speculate that our glaucoma prevalence was underestimated, even though disease incidence can be overestimated when using national claims data [10, 11].

Other population-based studies found a substantially higher prevalence of glaucoma than reported in the current study. Our study may have underestimated glaucoma prevalence for several reasons. First, the most common type of glaucoma is chronic asymptomatic disease. Because glaucoma can remain asymptomatic until it becomes severe, there is a high likelihood that the number of affected individuals is much higher than the number known to have the disease [12, 13]. In fact, several studies have reported a very low awareness of and knowledge about glaucoma in the general population [5, 14], with only 10–50% of people with glaucoma aware that they have the disease [12, 13, 15–17]. Additionally, the high rate of undiagnosed POAG (92.7%) was shown in a population-based study in Korea [18]. When we estimate the number of patients with glaucoma using the 3.5% glaucoma prevalence reported in a systematic review [2], the proportion of undetected patients with glaucoma in our study was about 70% (Table 5). Only patients already diagnosed with glaucoma were included in our analyses. Therefore, the chronic characteristics of glaucoma may be well reflected. Moreover, our underestimated prevalence of glaucoma may have resulted from the high proportion of NTG in Korea. Although elevated intraocular pressure (IOP) is the hallmark sign and a well-known risk factor for glaucoma, several population-based studies found that IOP was <22 mmHg in 25–50% of glaucoma patients [19]. In fact, 77% of patients with OAG in Korea have NTG [18]. Unfortunately, NTG cannot be detected through tonometry. Therefore, it is more difficult to detect than OAG with ocular hypertension.

Even though our estimated prevalence of diagnosed glaucoma was lower than in other population-based studies, the features of glaucoma prevalence were similar. In agreement with previous studies [2, 3, 18, 20], our multivariate analysis revealed a gender-adjusted OR for glaucoma of 1.7 with each decade increase in age. Additionally, glaucoma was more prevalent in males than in females after adjusting for age (OR: 1.1–1.3). Some controversy remains about whether gender affects glaucoma prevalence, but previous studies conducted in Korea have also shown a higher prevalence in males [6, 20]. In our multivariate analysis model, age had a more significant effect on glaucoma prevalence than gender (Table 3). Additionally, the fact that OAG and ACG accounted for the highest and second highest proportion of glaucoma cases, respectively, is consistent with other studies [2, 20, 21]. We found that the OAG/ACG ratio was 4.9–7.2 (Table 2), which is also similar to other studies conducted on East Asian populations [7, 21].

Both the number of glaucoma cases and the number of glaucoma surgeries showed an increasing trend over time. Interestingly, the proportion of money spent on glaucoma surgeries decreased annually (Figure 2). This may have been caused by a progressive increase in antiglaucoma medication usage.

Our study had several imitations. First, people with undiagnosed glaucoma were not included in our estimates of glaucoma prevalence, which may have resulted in an underestimated prevalence. Additionally, national claims data do not always match hospital chart records. It has been shown that national claims data and hospital records only have a 70–80% concordance rate [22]. Second, although data from the national claims database better represents the clinical situation than data from population-based studies, inconsistencies between clinicians in disease diagnostic criteria and practical patterns may have influenced our results, especially for angle closure glaucoma. Finally, we did not assess risk factors for glaucoma (e.g., myopia, elevated IOP, and comorbid conditions), which would require further investigation with additional national claims data.

In conclusion, the prevalence and socioeconomic burden of glaucoma are increasing annually. Nevertheless, based on national claims data, we speculate that many patients with glaucoma remain undiagnosed in Korea. Ways to identify these patients are needed so that the disease can be managed to minimize vision loss in glaucoma patients with a long life expectancy.

## Figures and Tables

**Table 1:** Glaucoma prevalence and glaucoma care costs between 2008 and 2013 in Koreans over 40 years of age.

	2008	2009	2010	2011	2012	2013
Total population	22,092,004	22,835,230	23,555,512	24,315,896	24,988,953	25,678,881
Population visiting eye clinic	6,958,082	7,547,494	8,360,447	8,984,627	9,284,879	9,615,033
Number of glaucoma patients (% glaucoma patients)	175,899 (2.53)	190,651 (2.53)	207,986 (2.49)	238,124 (2.65)	251,468 (2.71)	271,141 (2.82)
Male	86,534	93,940	103,066	117,578	123,814	133,914
Female	89,365	96,711	104,920	120,546	127,654	137,227
Glaucoma care costs (millions)	$16.5	$19.3	$21.7	$22.9	$27.2	$29.8
Annual glaucoma care costs per person	$93.6	$101.1	$104.3	$96.2	$108.0	$110.0

Results based upon data from the national claims database. The 2008–2013 Glaucoma care cost in 2013 constant dollars, using 3% average annual inflation rate and applying the annual KRW/USD exchange rate of each year.

**Table 2:** Estimated prevalence (%) of glaucoma in Koreans over 40 years of age.

	2008	2009	2010	2011	2012	2013
OAG (95% CI)	0.46 (0.456–0.462)	0.48 (0.473–0.478)	0.50 (0.494–0.500)	0.57 (0.570–0.577)	0.61 (0.603–0.609)	0.65 (0.648–0.654)
ACG (95% CI)	0.093 (0.092–0.094)	0.095 (0.094–0.096)	0.100 (0.099–0.101)	0.095 (0.094–0.096)	0.090 (0.090–0.091)	0.090 (0.089–0.091)
Overall (95% CI)	0.80 (0.793–0.800)	0.83 (0.831–0.839)	0.88 (0.879–0.887)	0.98 (0.975–0.983)	1.01 (1.002–1.010)	1.06 (1.052–1.060)
Overall (male/female)	0.82/0.78	0.86/0.82	0.91/0.86	1.00/0.96	1.03/0.99	1.08/1.03
Proportion of OAG	57.6%	56.9%	56.3%	58.6%	60.2%	61.7%
Proportion of ACG	11.7%	11.4%	11.3%	9.7%	8.9%	8.6%
OAG/ACG ratio	4.94	5.00	5.00	6.04	6.75	7.21

Data obtained from the national claims database. OAG = open angle glaucoma, ACG = angle closure glaucoma, and CI = confidence interval.

**Table 3:** Effect of age and gender on the prevalence of glaucoma.

		Univariate		Multivariate	
		Odds ratio (95% CI)	*P* value	Odds ratio (95% CI)	*P* value
2008	Age, per decade increase	1.664 (1.658–1.670)	<0.001	1.616 (1.610–1.622)	<0.001
	Sex		<0.001		<0.001
	Female	1.000 (reference)		1.000 (reference)	
	Male	1.047 (1.037–1.056)		1.131 (1.120–1.142)	

2009	Age, per decade increase	1.683 (1.678–1.689)	<0.001	1.759 (1.753–1.766)	<0.001
	Sex		<0.001		<0.001
	Female	1.000 (reference)		1.000 (reference)	
	Male	1.048 (1.038–1.057)		1.300 (1.288–1.311)	

2010	Age, per decade increase	1.704 (1.698–1.709)	<0.001	1.723 (1.717–1.729)	<0.001
	Sex		<0.001		<0.001
	Female	1 (reference)		1.000 (reference)	
	Male	1.058 (1.048–1.067)		1.237 (1.226–1.248)	

2011	Age, per decade increase	1.719 (1.714–1.725)	<0.001	1.738 (1.733–1.743)	<0.001
	Sex		<0.001		<0.001
	Female	1.000 (reference)		1.000 (reference)	
	Male	1.048 (1.039–1.056)		1.228 (1.218–1.238)	

2012	Age, per decade increase	1.712 (1.707–1.717)	<0.001	1.729 (1.724–1.735)	<0.001
	Sex		<0.001		<0.001
	Female	1.000 (reference)		1.000 (reference)	
	Male	1.040 (1.032–1.049)		1.214 (1.204–1.223)	

2013	Age, per decade increase	1.730 (1.725–1.735)	<0.001	1.748 (1.743–1.753)	<0.001
	Sex		<0.001		<0.001
	Female	1.000 (reference)		1.000 (reference)	
	Male	1.046 (1.039–1.054)		1.223 (1.214–1.232)	

CI = confidence interval.

**Table 4:** The cost and number of glaucoma surgeries (including laser procedures) in Korea between 2009 and 2013 (2008 database was unavailable).

	2009	2010	2011	2012	2013
Cost of glaucoma surgeries (US$)	2,226,234	2,148,179	2,211,149	2,414,895	2,847,254

Iridectomy (%)	5,362 (54.9%)	5,288 (55.4%)	5,469 (54.4%)	5,809 (53.2%)	6,202 (49.6%)
Trabeculectomy (%)	2,301 (23.4%)	2,152 (22.6%)	2,489 (24.7%)	3,067 (28.1%)	4,013 (32.1%)
Glaucoma implant surgery (%)	831 (8.5%)	946 (9.9%)	928 (9.2%)	991 (9.1%)	1,009 (8.1%)
Iris, ciliary photocoagulation	822	668	779	614	698
Trabeculotomy	236	279	206	205	365
Ciliary body cryotherapy	96	86	93	95	75
Nonpenetrating filtering surgery	63	47	31	38	58
Other filtering surgery	57	73	64	89	80
Total number of surgeries	9,773	9,541	10,061	10,912	12,502

Results based on data from the national claims database.

**Table 5:** Estimated prevalence of undetected glaucoma.

	2008	2009	2010	2011	2012	2013
Population visiting an eye clinic	6,958,082	7,547,494	8,360,447	8,984,627	9,284,879	9,615,033
Number of patients diagnosed with glaucoma (from national claims database)	175,899	190,651	207,986	238,124	251,468	271,141
Estimated number of glaucoma patients^a^	773,220	799,233	824,443	851,056	874,613	898,761
Estimated number of patients with undetected glaucoma^b^	529,687	535,071	531,827	536,594	549,643	562,235
Proportion of patients with undetected glaucoma	68.5%	66.9%	64.5%	63.0%	62.8%	62.5%

Results based upon data from the national claims database. ^a^Estimated value based on the 3.5% glaucoma prevalence reported in a systematic review [2] (i.e., total population × 0.035). ^b^Estimated value based on the population who had not been to an eye clinic within a year (i.e., 0.35 × [total population − population who visited an eye clinic within one year]).
